# Study on Novel Surface Defect Detection Methods for Aeroengine Turbine Blades Based on the LFD-YOLO Framework

**DOI:** 10.3390/s25072219

**Published:** 2025-04-01

**Authors:** Wei Deng, Guixiong Liu, Jun Meng

**Affiliations:** 1 School of Mechanical and Automotive Engineering, South China University of Technology, Guangzhou 510640, China; 202310180425@mail.scut.edu.cn; 2 Jetech Technology Company, Shenzhen 518102, China; mengjun@jeettech.com

**Keywords:** ATB, defect detection, LDconv, DAT, Focaler-CIoU

## Abstract

This study proposes a novel defect detection method to address the low accuracy and insufficient efficiency encountered during surface defect detection on aeroengine turbine blades (ATBs). The proposed approach employs the LDconv model to adjust the size and shape of convolutional kernels dynamically, integrates the deformable attention mechanism (DAT) to capture minute defect features effectively, and uses Focaler-CIoU to optimize the bounding box loss function of the detection network. Our approaches collectively provide precise detection of surface defects on ATBs. The results show that the proposed method achieves a mean average precision (*mAP*_0.5_) of 96.2%, an F-measure of 96.7%, and an identification rate (*I_r_*) of 98.8%, while maintaining a detection speed of over 25 images per second. The proposed method meets the stringent requirements for accuracy and real-time performance in ATB surface defect detection.

## 1. Introduction

The aeroengine, a critical aircraft component, relies heavily on the quality of its turbine blades (ATBs) to ensure optimal performance and safety [1]. During operation, the engine is exposed to extreme conditions, including high temperatures, high pressures, and rotational speeds. In addition, the blades endure thermal stress, deformation forces, vibrational loads, aerodynamic forces, and centrifugal forces equivalent to 2000 times their weight during engine startup, speed changes during operation, and shutdown. These factors make ATBs highly susceptible to damage and failure, resulting in regular inspections to ensure their reliability and safety [2]. However, the large number and varying geometries of aeroengine blades pose significant challenges for defect detection. Traditional inspection methods are often time-consuming, labor-intensive, and not very reliable. Current visual inspection techniques for ATB defects can be broadly categorized into two different approaches: traditional manual inspection and deep learning-based visual inspection. Traditional methods strongly rely on human judgment, often guided by subjective assessments or referenced in aeroengine maintenance manuals. These approaches are inefficient [3,4] and susceptible to errors such as missed or false detections, compounded by inspector fatigue. In contrast, deep learning-based visual inspection methods leverage advanced endoscopic technology and artificial intelligence. By employing convolutional neural networks (CNNs) to extract features and using forward and backward propagation for defect detection and recognition, compared to traditional detection methods, the application of deep learning methods has significantly improved the efficiency and accuracy of ATB defect detection, while also enabling intelligent and automated detection processes [5,6,7]. In recent years, numerous researchers have continued to conduct in-depth studies on deep learning methods for ATB defect detection, making it a prominent research focus [8,9]. Meanwhile, in the field of surface defect detection, significant progress has also been made with deep learning methods based on various architectures. For example, Huang et al. [10] proposed SSA-YOLO, which significantly improved the detection accuracy of tiny defects on hot-rolled strip steel surfaces by introducing a channel attention mechanism. Cui et al. [11] developed SDDNet, combining feature retention blocks (FRB) and skip dense connection modules (SDCM) to address challenges such as large variations in defect textures and small defect sizes, achieving high-speed and high-precision surface defect detection. Usamentiaga et al. [12] systematically reviewed deep learning-based methods for metal surface defect detection, highlighting the advantages of the YOLO series algorithms in balancing real-time performance and accuracy.

However, the existing YOLO improvement methods are primarily designed for general industrial scenarios and face limitations when addressing the specific requirements of aeroengine turbine blade (ATB) surface defect detection, such as the diverse shapes and textures of damage and the presence of tiny defects. These limitations include the following: the insufficient adaptability of fixed-size convolution kernels, making it difficult to capture features of tiny defects on ATB surfaces; traditional IoU loss functions tend to optimize bounding boxes for larger targets, leading to reduced localization accuracy for tiny defects; the downsampling operations in YOLO reduce the resolution of deep feature maps, further exacerbating the dilution of features for tiny defects. These issues severely constrain the performance of YOLO in ATB surface defect detection and serve as the core motivation for the improvements proposed in this paper. To address the aforementioned challenges, we propose a novel surface defect detection method for ATBs based on the LFD (LDconv + Focaler-CIoU + DAT)-YOLO framework, which integrates YOLOv8, linear deformable convolution (LDconv), Focaler-CIoU, and the deformable attention mechanism (DAT). The proposed approach involves three innovative key approaches: replacement of the CBS module with Ldconv, optimization of the bounding box loss function, and integration of the deformable attention mechanism. Collectively, the proposed LFD-YOLO framework improves the level of accuracy and robustness of ATB defect detection, resulting in precise, in-situ, and automated inspection. It also ensures real-time performance, making it highly suitable for industrial applications.

The contributions of this paper are summarized as follows:

- Proposal of an LFD-YOLO-based ATB surface defect detection method: a novel method for detecting surface defects on ATBs is proposed, employing the LFD-YOLO framework. This approach incorporates LDconv to adjust the size and shape of convolutional kernels dynamically, utilizes the deformable attention mechanism to learn minute defect features effectively, and includes the Focaler-CIoU module to optimize the bounding box loss function of the network. These innovations collectively provide precise and accurate detection of surface defects on ATBs.
- Construction of an ATB detection framework: a specialized ATB detection framework is developed to capture defect images, including cracks, nicks, dents, and burns. The collected dataset is used to train the LFD-YOLO network, ensuring robust performance in defect identification.
- Experimental validation: extensive experimental data demonstrate the effectiveness of the proposed method. The LFD-YOLO framework achieves a mean average precision (*mAP*_0.5_) of 96.2%, an F-measure of 96.7%, and an identification rate (*I_r_*) of 98.8%. The system processes over 25 images per second, meeting the stringent requirements for accuracy and real-time performance in ATB surface defect detection.

## 2. Background Information

### 2.1. YOLO Series Network for ATB Surface Defect Detection

The YOLO (You Only Look Once) series of deep learning-based detection and recognition algorithms employ an independent convolutional neural network architecture to simultaneously classify objects and regress bounding box coordinates within the image. Known for their real-time performance, simplicity, efficiency, high accuracy, and strong generalization capabilities, these algorithms have been widely applied in turbine blade defect detection and other related areas [13,14,15,16,17,18,19,20,21]. For instance, Chen et al. [22] proposed a YOLOv4-based defect detection model for turbine blades. Their model utilizes spatial pyramid pooling (SPP) and the path aggregation Network (PANet) as their parameter aggregation methods [23]. By incorporating an SPP module, a CSPDarknet53 backbone, and a PAN, the YOLOv4 architecture can be optimized for rapid defect detection. Experimental data from a self-constructed blade defect dataset have demonstrated a mean average precision (mAP) of 0.9. Li et al. [24] introduced the intelligent defect detection model DDSC-YOLOv5s. This model integrates a deformable convolutional network to enhance feature extraction efficiency through depthwise separable convolution. K-means clustering is employed to optimize anchor box sizes, which improves the real-time detection capability for ATB defects. Experimental data from a self-built borescope blade defect dataset yield an mAP of 0.838, with only a 7.9% increase in computational load compared to the original YOLOv5. Liao et al. [25] developed an optimized YOLOv5 model with a BiFPN (Bidirectional feature pyramid network) structure for detecting and classifying defects on Si3N4 turbine blades. Experimental data from an enhanced dataset of blade X-ray defect images achieve an mAP of 0.986, with the detection speed as low as 16 milliseconds. In [26], a DBFF-YOLOv4-based algorithm is proposed to address the challenges of error-prone and time-consuming X-ray defect detection in turbine blades. This approach uses dual backbones for hierarchical defect feature extraction and introduces a novel connection method that incorporates all feature maps. The resulting defect detection system achieves an identification rate (*I*_r_) of 96.7% on a self-constructed blade defect dataset. In [27], an optimized YOLOv7 model incorporating a convolutional block attention module and Alpha_GIOU (CBAM) is proposed. The dataset is preprocessed using gamma correction, and an improved CBAM is embedded into the model backbone. A branch is added to the CBAM channel attention module, and Alpha_GIOU replaces CIoU as the loss function. These modifications and additions significantly improve the real-time performance and visualization of ATB defects. Experimental work on a self-built blade defect dataset demonstrates an mAP of 0.961, representing a 1.0% improvement over the original YOLOv7 model.

As described above, the YOLO algorithm series employs a global convolutional neural network to process entire images, generating an efficient single-stage detection pipeline. These algorithms have been extensively applied in turbine blade defect detection due to their robustness and real-time performance. This study adopts and further optimizes the YOLO series framework to achieve precise and efficient surface defect detection for ATBs.

### 2.2. Other Common Networks Used for ATB Surface Defect Detection

In addition to the YOLO series networks, other deep learning networks such as Faster R-CNN [28,29], Mask R-CNN [30], and VFNet [31] have also been studied for ATB defect detection. In [32], a modified Faster R-CNN-based model was proposed to address the issues of tiny and sometimes discontinuous defects in blade surface defect detection. This model replaces RoI pooling with RoI align, incorporates feature pyramid networks (FPN) combined with ResNet50 for feature extraction, and improves the non-maximum suppression (NMS) algorithm. The model was tested on a self-constructed blade defect dataset, achieving a mean average precision (*mAP*) of 0.79, a 17.5% improvement over the original Faster R-CNN. Shang et al. [33] developed an enhanced Mask R-CNN-based method for ATB surface damage detection. This method constructs an enhanced Mask R-CNN network with three functionalities: damage pattern classification, damage localization, and damage region segmentation. It focuses on shallow texture information through a texture-focused multiscale feature fusion network (TFNet) [34] and introduces a balanced L1 loss (BL) to improve localization accuracy. Experimental data on a self-constructed simulated blade defect image dataset yielded an *mAP* of 0.604. Liu et al. [35] proposed a tiny defect detection network (MVFNet), which builds upon VFNet by using a spatial feature pyramid network (SFPN) for feature fusion. They introduced a spatial attention residual module (SAR) into the SFPN to capture global spatial information, thereby improving the detection accuracy of tiny defects on blade surfaces. Further tests on a blade defect dataset resulted in an *mAP* of 0.735, 7.7% higher than the original VFNet.

It is evident that networks such as Faster R-CNN, Mask R-CNN, and VFNet each have their unique characteristics and have been selected for use in research areas such as industrial defect detection. Compared to two-stage detection methods, the one-stage detection approach of the YOLO series significantly enhances detection speed by simplifying the object detection task into a single forward propagation, while maintaining high accuracy. This characteristic makes YOLO particularly suitable for ATB surface defect detection tasks, as this detection scenario places extremely high demands on real-time performance, requiring rapid response while ensuring detection accuracy.

The Experimental and Results sections are organized as follows: Section 3 describes the LFD-YOLO framework in detail. Section 4 presents the experimental setup, including the experimental data, to demonstrate the superior performance of the LFD-YOLO model, while Section 5 highlights the most significant advantages of the proposed model and its limitations.

## 3. Mechanism of ATB Surface Defect Detection Based on LFD-YOLO

### 3.1. Overall Framework for ATB Surface Defect Classification and Detection

**Figure 1:**
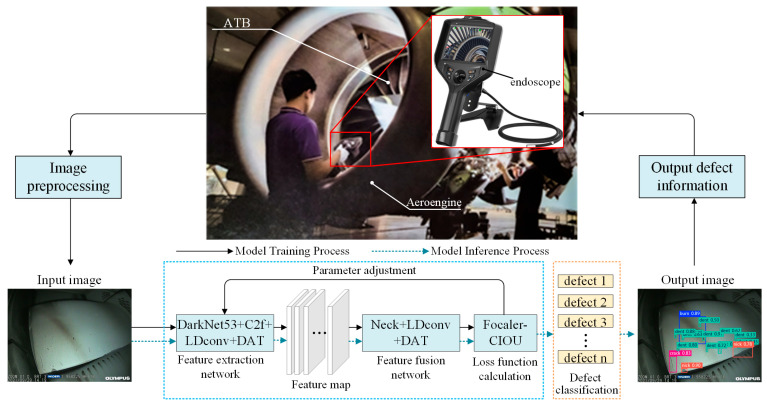
Overall framework diagram of the ATB surface defect detection algorithm based on LFD-YOLO.

Figure 1 illustrates the overall framework for ATB surface defect detection based on LFD-YOLO, which includes the following key modules: image preprocessing, feature extraction network, feature fusion network, loss function computation, defect classification, and defect information output. Specifically: (1) the image preprocessing module is designed to preprocess the input raw images by adjusting image attributes for enhanced visibility of defect features; (2) the DarkNet53 + C2f + LDconv + DAT feature extraction network is employed to extract morphological and positional defect features, as well as high-level semantic information, and outputs feature maps at different scale levels; (3) the Neck + LDconv + DAT feature fusion network utilizes the deformable attention mechanism to dynamically select sampling points in the image, focusing intensively on critical defect regions (this approach significantly reduces computational load while maintaining robust performance); (4) the Focaler-CIoU loss function computation module optimizes the IoU loss for multicategory regression samples using a linear interval mapping method, thereby improving the regression accuracy of the network; (5) the defect classification module classifies and displays the different types of ATB defects; (6) the defect information output module is used to output surface defect information related to ATBs.

**Figure 2:**
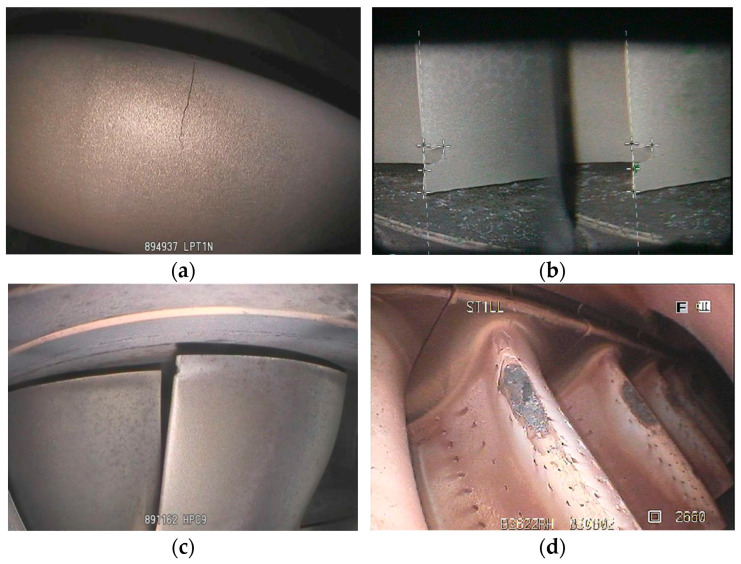
Images of typical surface defects in ATBs with high detection demands: (**a**) a crack, (**b**) nicks, (**c**) a dent, and (**d**) burns.

Under harsh working conditions, ATBs are prone to various defects such as cracks, notches, dents, burns, tears, curled edges at the blade tip, material loss/chips, burn-through, bending, bulges, deposits, overlaps/laps, corrosion, carbon buildup, fractures, sulfidation, and coating loss. Among these, defects that require highly accurate detection include cracks, notches, dents, and burns [36]. Figure 2 presents typical surface defects in ATBs that require good detection methods; Figure 2a–d represent cracks, nicks, dents, and burns, respectively.

### 3.2. Design of an LFD-YOLO Network for ATB Surface Defect Detection

**Figure 3:**
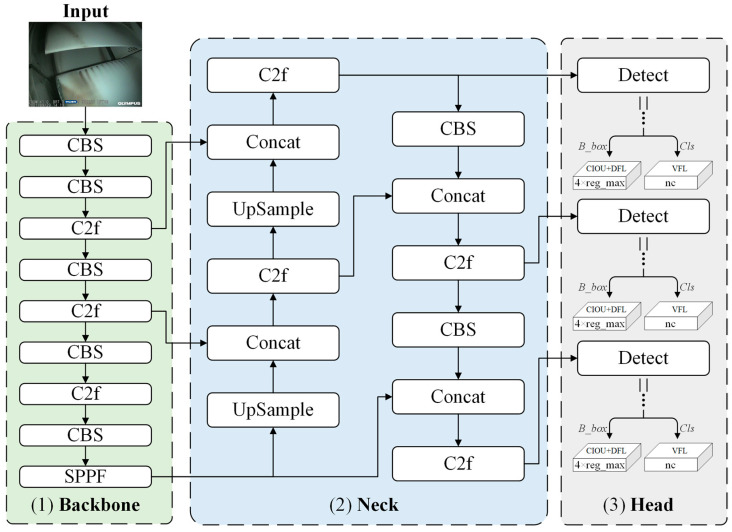
Network architecture of YOLOv8 [19].

Figure 3 illustrates the network architecture of YOLOv8 [19], which consists of four main parts: input, backbone, neck, and head. Specifically, the input section employs mosaic-type data augmentation for images fed into the network, including adaptive image scaling, grayscale filling, and other preprocessing techniques; the backbone section comprises structures such as CBS, C2f, and SPPF, which extract image features through convolution and pooling operations; the Neck section utilizes the PAN–FPN structure, achieving feature map fusion at different scales via up-sampling, down-sampling, and feature concatenation; and the decoupled head structure of the head section decouples classification and regression processes. It includes positive and negative sample matching and loss computation and obtains the detection targets’ category and location information based on feature maps at different scales.

**Figure 4:**
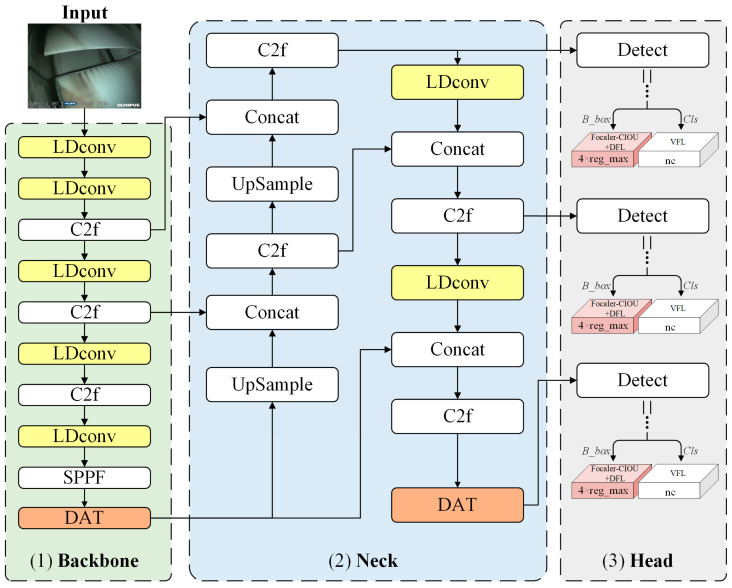
Network architecture of LFD-YOLO.

Based on YOLOv8 as the benchmark network, this paper proposes a novel network structure defined as LFD-YOLO (Figure 4). Unlike the classic YOLOv8 network, the LFD- YOLO network replaces the original CBS module with the LDconv module in the backbone section. It incorporates the deformable attention mechanism to improve the detection capability for tiny defects. In the neck section, the PAN–FPN structure is combined with the LDconv + DAT deformable attention mechanism to fuse multiscale features and improve the detection of various types of ATB surface defects. The target category and location information are obtained from feature maps at different scales. The head section employs Focaler-CIoU to optimize the bounding box loss function, which improves the accuracy of bounding box regression.

The following sections describe the mechanisms and characteristics of three core innovations: the introduction of LDconv to replace CBS, the addition of the DAT deformable attention mechanism, and the optimization of the bounding box loss function using Focaler-IoU.

#### 3.2.1. Replacement of CBS by LDconv

Convolution operations are widely used in deep learning and other fields for extracting image features. However, standard convolution operations have several limitations: they are confined to local windows, making it difficult to capture information from other locations; moreover, the fixed shape of convolution kernels results in a parameter count that is proportional to the kernel size [37]. To address these issues, this paper employs a linear deformable convolution (LDConv) algorithm [38] that replaces the CBS module containing standard convolution in the YOLOv8 network (as shown in the light-yellow modules in the backbone and neck sections of Figure 4). LDConv provides an arbitrary number of parameters and sampling shapes for the convolution kernels, which enables adaptation to target variations, reduces network overhead, and improves the detection of tiny defects.

**Figure 5:**
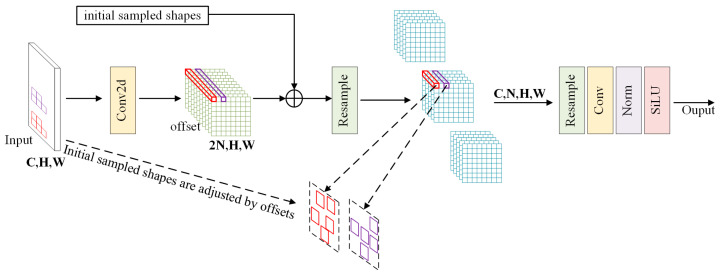
Diagram of the LDconv structure [38].

Figure 5 illustrates the structure of LDconv (using a kernel size of *N* = 5 as an example) [38]. First, the initial sampling coordinates *P*_0_ for the convolution kernel need to be generated. Algorithm 1 presents the Python 3.11 pseudocode for the *P*_0_ generation algorithm [38]. Regular or irregular coordinates are generated by calculating the base integers of the convolution kernel, followed by coordinate concatenation and other steps to obtain the corresponding sampling coordinates (*P*_0_ + *P_i_*) for any point *P_i_* on the feature map. For the sampling grid *R* and convolution parameters *w*, the corresponding convolution operation is expressed as follows:

(1) $$Conv\left(P_{i}\right)=\sum_{P_{0}\in R}w\times \left(P_{0}+P_{i}\right)$$

Next, a Conv2d convolution operation is performed on the input image, from which the kernel offset *C*_offset_ is derived. This offset is then used to adjust the original coordinates, enabling dynamic reshaping of the convolution kernel. Based on the adjusted sampling shape, the feature map undergoes resampling (resample). The resampled feature map is then reshaped, convolved again, normalized, and finally passed through the SiLU activation function to output the resulting feature map.
**Algorithm 1** Pseudocode for Initial Coordinate Generation for Convolution Kernels in a PyTorch-like Approach [38]# func get_p_o(num_param, dtype) # num_param:the kernel size of LDConv # dtype:the type of data ####### function body ######## # get a base integer to define coordinate base_int = round(math.sqrt(num_param)) row_number = num_param//base_int mod_numer = num_param % base_int # get the sampled coordinate of regular kernels p_o_x,p_o_y = torch.meshigrid( torch.meshgrid(0, row_numb) torch.meshgird(0, base_int)) # flatten the sampled coordinate of regular kernels p_o_x = torch.flatten(p_o_x) P_o_y = torch.flatten(p_o_y) # get the sampled coordinate of irregular kernels If mod_number > 0: mod_p_o_x, mod_p_o_y = torch.meshgird( torch.arange(row_number,row_number+1,torch.arange(0,mod_number)) mod_p_o_x = torch.flatten(mod_p_o_x) mod_p_o_y = torch.flatten(mod_p_o_y) P_o_x,p_o_y = torch.cat((p_o_x,mod_p_o_x)),torch.cat((p_o_y,mod_p_o_y)) # get the completed sampled coordinate p_o = torch.cat([p_o_x, p_o_y], 0) p_o = p_o.view(1, 2 * num_param, 1, 1).type(dtype) return p_o

#### 3.2.2. Incorporation of the DAT Deformable Attention Mechanism

Some defects on the ATB surface are small but occur in high numbers. Using conventional attention mechanisms to process all pixels in the image would result in high computational costs. To address this, we introduce the deformable attention transformer mechanism to optimize the feature extraction and feature fusion networks of YOLOv8. DAT dynamically selects sampling points, which allows the network to focus on important features, thereby reducing computational load while improving the accuracy of ATB surface defect detection [39]. By adding DAT to the final layer of the backbone feature extraction network (as shown in the orange module in the backbone section of Figure 4), the network can flexibly control the intensity and scope of attention based on the input image features and better capture subtle features. In addition, the network’s ability to perceive and fuse deep discriminative features and high-level semantic information is significantly improved by incorporating DAT after the C2f module in the down-sampling stage of the neck feature fusion network (as shown in the orange module in the neck section of Figure 4).

**Figure 6:**
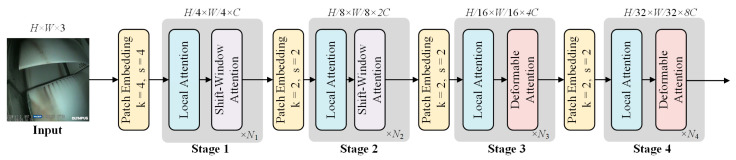
Structure of the DAT [39].

Figure 6 illustrates the structure of the deformable attention transformer [34]. The DAT architecture adopts a 4-stage hierarchical framework. As the stages progress, the spatial dimensions of the feature maps are split in half, while the number of channels doubles. During the initialization phase, a 4 × 4 convolution is applied to reduce the image size to one-fourth of its original dimensions, thereby reducing the computational load. In stages 1–2, local information is integrated with shift-window attention to better capture local features. In stages 3–4, the deformable attention (DA) module is added to simulate the relationship between local and global contexts. This type of structure balances the need for local and global recognition, thus effectively reducing computational costs while improving both accuracy and efficiency.

**Figure 7:**
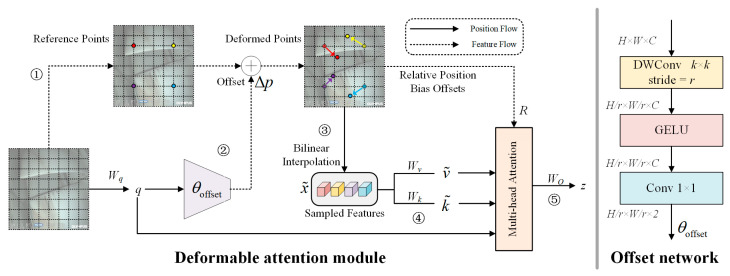
Flowchart of the DA module [39].

Figure 7 illustrates the flowchart of the deformable attention (DA) module. The process includes the following steps:

(1) For an input feature map *x* with dimensions *H* × *W* × *C*, the predefined scaling factor is α. Then, the down-sampling expression for the original grid can be represented as follows (as indicated by the process labeled ① in Figure 7):

(2) $$H_{G}=\frac{H}{\alpha },\;W_{G}=\frac{W}{\alpha }$$

From the down-sampled *H_G_* × *W_G_* × 2 uniform grid, the original reference points *P*_re_ are selected, where the values of the reference points are linearly spaced coordinates {(0, 0), …, (*H_G_* − 1, *W_G_* − 1)}.

(2) If the projection weight for the query is defined as *W_q_*, then by linearly projecting the feature map *x* onto the query tokens, the query vector *q* is obtained. Then, through the offset generation subnetwork (offset network), the deformation offset Δp is generated (as indicated by the process labeled ② in Figure 7):

(3) $$\Delta p=\theta_{\mathrm{offset}}(q),\;q=xW_{q}$$

The deformed reference points *P*_de_ are calculated by combining the original reference points *P*_re_ and the offset Δp. Bilinear interpolation is then applied to *P*_de_ to obtain the sampled feature map $\tilde{x}$ from *x* (as indicated by the process labeled ③ in Figure 7):

(4) $$\tilde{x}=\phi (x;P_{\mathrm{de}}),\;P_{\mathrm{de}}=P_{re}+\Delta p$$

(3) The vectors $\tilde{k}$ and $\tilde{v}$ are obtained by performing linear projections on $\tilde{x}$ in the directions of the key tokens and value tokens, with projection weights *W_k_* and *W_v_*, respectively (as indicated by the process labeled ④ in Figure 7):

(5) $$\tilde{k}=\tilde{x}W_{k},\;\tilde{v}=\tilde{x}W_{v}$$

(4) The multi-head attention module integrates the outputs. If the softmax function is denoted as σ(·), then the head size of the multi-head attention is *d*_head_, and the query, key, and value vectors for the mth attention head are $q^{\left(m\right)}$, ${\tilde{k}}^{\left(m\right)}$, and ${\tilde{v}}^{\left(m\right)}$, respectively, the bilinear interpolation operation for the relative position bias is denoted as $\phi (\hat{B};R)$, and the output *z*^(*m*)^ of the multi-head attention can be expressed as follows (as indicated by the process labeled ⑤ in Figure 7):

(6) $$z^{\left(m\right)}=\sigma \left(q^{\left(m\right)}{\tilde{k}}^{(m)⊤}/\sqrt{d_{\mathrm{head}}}+\phi (\hat{B};R)\right){\tilde{v}}^{\left(m\right)}$$

#### 3.2.3. Optimization of the Focaler-IoU Bounding Box Loss Function

In defect detection, the intersection over union (IoU) is commonly used as a critical parameter to evaluate the performance of detection networks [13]. If the predicted bounding box and the ground-truth bounding box are denoted as *B* and *B^gt^*, respectively, the IoU can be calculated using the following equation:

(7) $$IoU=\frac{\left|B\cap B^{gt}\right|}{\left|B\cup B^{gt}\right|}$$

The IoU serves as the criterion for distinguishing positive and negative samples during the training phase and as the loss function for the detection network. It is also utilized in non-maximum suppression (NMS) during the inference stage. However, when the IoU equals zero, it becomes difficult to accurately characterize the positional relationship between two bounding boxes, leading to stalled gradient updates and hindering further learning and optimization of the network.

The traditional CIoU loss function exhibits significant bias toward large targets, resulting in insufficient localization accuracy for small defects. This bias arises because CIoU assigns equal weight to all targets, neglecting the optimization for smaller ones. To address this issue, we introduce Focaler-CIoU. In the YOLOv8 network, the CIoU is employed as the bounding box regression loss. If the Euclidean distance between the center points of *B* and *B^gt^* is defined as $\rho^{2}\left(B,B^{gt}\right)$, the weight parameter is ε, the aspect ratio correction factor is *v*_AR_, the diagonal distance of the minimum bounding box enclosing *B* and *B^gt^* is *c*_MBB_, the width and height of *B* are *w^B^* and *h^B^*, and the width and height of *B^gt^* are defined as *w^B−gt^* and *h^B−gt^*, respectively, the CIoU and its loss function *L*_CIoU_ can be calculated using the following expressions:

(8) $$\left\{\begin{array}{l} \mathrm{CIoU}=\mathrm{IoU}-\frac{\rho^{2}\left(B,B^{gt}\right)}{{c_{MBB}}^{2}}-\varepsilon v_{AR};\;L_{CIoU}=1-\mathrm{CIoU}\\ \varepsilon =\frac{v_{AR}}{(1-\mathrm{IoU})+v_{AR}};\;v_{AR}=\frac{4}{\pi^{2}}{\left(\arctan\frac{w^{B-gt}}{h^{B-gt}}-\arctan\frac{w^{B}}{h^{B}}\right)}^{2} \end{array}\right.$$

To better focus on different regression samples and improve the performance of the detection network across various detection tasks, this study replaced CIoU in YOLOv8 with IoU^Focaler^ [40]. The equation for IoU^Focaler^ is as follows:

(9) $${\mathrm{IoU}}^{Focaler}=\left\{\begin{array}{ll} 0, & \mathrm{IoU}<d_{Focaler}\\ \frac{\mathrm{IoU}-d_{Focaler}}{u_{Focaler}-d_{Focaler}}, & d_{Focaler}\ll \mathrm{IoU}\ll u_{Focaler}\\ 1, & \mathrm{IoU}>u_{Focaler} \end{array}\right.$$

where $\left[d_{Focaler},u_{Focaler}]\in [0,\;1\right]$. By adjusting the values of $d_{Focaler}$ and $u_{Focaler}$, IoU^Focaler^ is a better fit for different regression samples. Therefore, the Focaler-IoU bounding box optimization loss function *L*_Focaler-CIoU_ proposed in this paper is expressed by Equation (10), which, after combining it with the DFL bounding box loss function *L_DFL_*, gives the optimized bounding box regression loss function *L*_B_box_ as follows:

(10) $$L_{\mathrm{Focaler}-\mathrm{CIoU}}=L_{\mathrm{CIoU}}+\mathrm{IoU}-{\mathrm{IoU}}^{\mathrm{Focaler}},\;L_{B\_\mathrm{box}}=L_{DFL}+L_{\mathrm{Focaler}-\mathrm{CIoU}}$$

The effectiveness and extent of these improvements described in the above sections were validated in subsequent experiments, as presented in the following section.

## 4. Experimental Design and Results Analysis

### 4.1. Experimental Design

**Figure 8:**
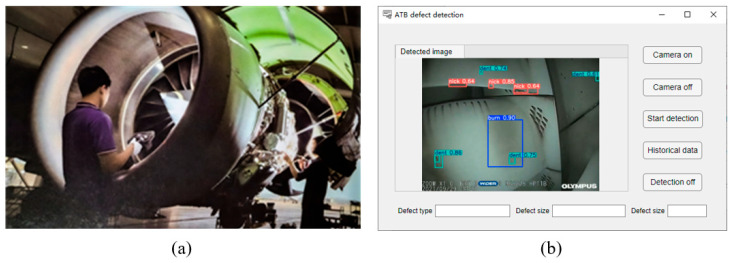
Diagram of the ATB surface defect detection system: (**a**) imaging process, (**b**) system interface.

Figure 8 illustrates the ATB surface defect detection system developed in this study. The system hardware includes an endoscope (OLYMPUS Corporation, Beijing, China) and a deep learning computer equipped with an AMD 5950x CPU and an NVIDIA GeForce RTX 3090 24 GB GPU. The software is designed to display surface defect information of ATBs.

The experimental dataset comprises 600 images (with a resolution of 1024 × 768 pixels), captured under the lighting conditions provided by the endoscope’s built-in lighting system to minimize background noise. The images include four types of defects on ATB surfaces: cracks, nicks, dents, and burns. The distribution of these defects in the dataset is as follows: cracks (20%), nicks (20%), dents (30%), and burns (30%). To enhance the diversity and robustness of the dataset, data augmentation techniques were employed, including random rotation (±30°), scaling (0.8× to 1.2×), horizontal and vertical flipping, and brightness adjustment (±20%). The dataset is divided into a training set of 480 samples, and validation and test sets of 60 samples each. The initial learning rate is set to 0.01, the batch size to 8, and the number of training epochs to 500. The ATB surface defect detection network is trained using the PyTorch 2.0.1 framework to identify and localize defects.

The experimental work employs *mAP*_0.5_, F-measure, recognition rate (*I_r_*), and FPS as evaluation metrics for detection performance, where:

(1) *mAP*_0.5_ is defined as the mean average precision at an IoU threshold of 0.5 [41]:

(11) $$mAP_{0.5}=\frac{\sum_{i=1}^{k}AP_{0.5}^{i}}{k},$$

where *k* is the number of categories and $AP_{0.5}^{i}$ represents the average precision of the *i*th category at an IoU threshold of 0.5.

(2) F-measure is defined as the weighted harmonic mean of precision and recall [42]:

(12) $$F-measure=\frac{(1+\alpha_{pr})\times Precision\times Recall}{\alpha_{pr}\times Precision+Recall},$$

where *α_pr_* represents the weighting coefficient between precision and recall. In this experiment, *α_pr_* is set to 0.8.

(3) *I_r_* denotes the recognition rate, defined as follows [41]:

(13) $$I_{r}=\frac{N_{\mathrm{defect}}}{M_{\mathrm{defect}}},$$

where *N*_defect_ and *M*_defect_ represent the number of correctly detected defects and the total number of defects in the sample images, respectively.

(4) FPS represents the number of images detected by the network per second [13]:

(14) $$FPS=\frac{Num_{\mathrm{frame}}}{Time}$$

where *Num*_frame_ is the total number of detected images and *Time* is the detection time. Real-time performance is one of the critical metrics for industrial inspection systems. In this study, based on the practical requirements of ATB defect detection, we define real-time performance as the system’s ability to process no fewer than 24 FPS.

### 4.2. Experimental Data Analysis of the LFD-YOLO Network Performance

#### 4.2.1. Comparative Experimental Data on the Network Performance

**Figure 9:**
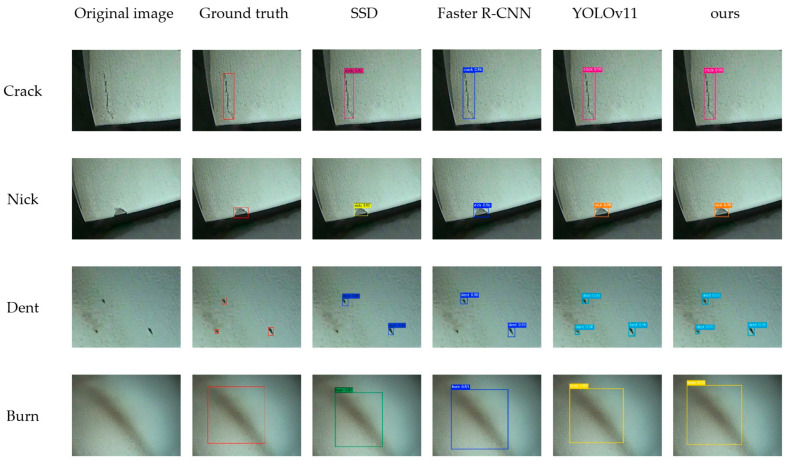
Comparison of prediction results across different networks.

To validate its effectiveness, we quantitatively compared the proposed LFD-YOLO network with other classical object detection networks, including SSD [43], Faster R-CNN [28], Mask R-CNN [30], YOLOv3 [44], YOLOv5 [16], YOLOv7 [18], YOLOv7-tiny [45], YOLOv8 [19], and YOLOv11 [46]. Among them, SSD is a classic model of single-stage detection methods, offering high detection speed and acceptable accuracy. Faster R-CNN and Mask R-CNN, as representatives of two-stage detection methods, are widely used in the field of object detection. Their high precision makes them important references for comparative experiments. The YOLO series models, with continuous iterations, have shown improved detection performance and are also included as references in comparative experiments. Table 1 presents the quantitative analysis results of these networks. Figure 9 illustrates a comparison of prediction results across different networks (with SSD and Faster R-CNN selected as representatives of single-stage and two-stage networks, respectively, and YOLOv11 chosen as a representative of the YOLO series, compared against our LFD-YOLO network).

The following information can be extracted from the data presented in Table 1:

(1) The SSD network achieved an FPS of 35.6 frames per second (f·s^−1^), demonstrating good real-time performance. However, its *mAP*_0.5_ of 61.2% and F-measure of 60.9% indicate relatively low accuracy during ATB defect detection. Similarly, the YOLOv7-tiny network achieved an FPS of 47.8 f·s^−1^, showing excellent real-time performance, but its *mAP*_0.5_ of 77.1% and F-measure of 76.8% suggested suboptimal ATB defect detection accuracy.
(2) The Faster R-CNN, Mask R-CNN, YOLOv3, YOLOv5, YOLOv7, YOLOv8, and YOLOv11 networks achieved *mAP*_0.5_ values of 73.7%, 76.3%, 77.5%, 86.8%, 89.1%, 89.7%, and 91.4%, respectively, and F-measure values of 74.1%, 78.2%, 76.7%, 86.9%, 88.7%, 90.3%, and 91.9%, respectively. These networks exhibited moderate to good real-time performance and reached a relatively high level of accuracy for ATB surface defect detection.
(3) The LFD-YOLO network achieved an FPS of 25.4 f·s^−1^ and an *mAP*_0.5_ of 96.2%, demonstrating the highest accuracy for ATB defect detection while meeting real-time performance requirements, showcasing significant advantages.

In summary, the proposed LFD-YOLO network for ATB surface defect detection outperforms other classical networks in terms of accuracy.

#### 4.2.2. Ablation Study

Ablation study I: to validate the effectiveness of Focaler-CIoU in ATB detection, an ablation study was conducted based on the improvements of YOLOv8 + LDconv + DAT, comparing it with EIoU [47], WIoU [48], and MPDIoU [49]. Table 2 presents the performance metrics comparison for ablation study I.

From Table 2, it can be observed that Focaler-CIoU performs exceptionally well in ATB defect detection. The LFD-YOLO network achieves superior *mAP*_0.5_, F-measure, and *I_r_* metrics compared to EIoU, WIoU, and MPDIoU. In terms of real-time performance, the four IoU improvements—EIoU, WIoU, MPDIoU, and Focaler-CIoU—have varying degrees of impact on the network, but all still meet the real-time performance requirements for ATB defect detection.

Ablation study II: to verify the optimal placement of the DAT module, based on the improvements of YOLOv8 + LDconv + Focaler-CIoU, the DAT module was separately added to the end of the backbone and the neck, corresponding to YOLOv8 + LDconv + Focaler-CIoU + DAT (backbone) and YOLOv8 + LDconv + Focaler-CIoU + DAT (neck) in the table. An ablation study was conducted by comparing these configurations with the LFD-YOLO network. Table 3 presents the performance metrics comparison for ablation study II.

The proposed LFD-YOLO network achieves improvements in *mAP*_0.5_, F-measure, and *I_r_* by 1.5%, 1.8%, and 3.6%, respectively, compared to adding the DAT module solely to the end of the backbone (94.7% → 96.2%, 94.9% → 96.7%, 95.2% → 98.8%). Similarly, improvements of 1.1%, 2.2%, and 2.8% are observed compared to adding the DAT module solely to the end of the neck (95.1% → 96.2%, 94.5% → 96.7%, 96.0% → 98.8%). These results demonstrate that adding the DAT module to both the backbone and the neck achieves the best performance, effectively enhancing the accuracy of ATB surface defect detection.

Ablation study III: to validate the effectiveness of each module in the proposed LFD-YOLO, an ablation study was conducted based on the YOLOv8 network. Table 4 presents the performance metrics of LFD-YOLO obtained from the ablation experiments.

From the results shown in Table 2, we obtained the following information:

(1) The YOLOv8 + LDconv network shows improvements in *mAP*_0.5_, F-measure, and *I_r_* by 3.1%, 2.9%, and 5.1%, respectively (89.7% → 92.8%, 90.3% → 93.2%, 87.9% → 93.0%), while the FPS decreases by 1.9 f·s^−1^ (29.1 f·s^−1^ → 27.2 f·s^−1^). This indicates that replacing CBS with LDconv effectively improved the accuracy of ATB surface defect detection, with a minor impact on real-time performance.
(2) The YOLOv8 + DAT network demonstrates improvements in *mAP*_0.5_, F-measure, and *I_r_* by 4.8%, 4.9%, and 8.4%, respectively (89.7% → 94.5%, 90.3% → 95.2%, 87.9% → 96.3%), while the FPS decreases by 1.5 f·s^−1^ (29.1 f·s^−1^ → 27.6 f·s^−1^). This suggests that incorporating the DAT deformable attention mechanism significantly increases the accuracy of ATB surface defect detection with a minimal impact on real-time performance.
(3) The YOLOv8 + Focaler-CIoU network achieves improvements in *mAP*_0.5_, F-measure, and *I_r_* by 2.4%, 1.2%, and 3.8%, respectively (89.7% → 92.1%, 90.3% → 91.5%, 87.9% → 91.7%), while the FPS decreases by 1.0 f·s^−1^ (29.1 f·s^−1^ → 28.1 f·s^−1^). This trend indicates that optimizing the bounding box loss function with Focaler-IoU moderately improved the accuracy of ATB surface defect detection with negligible impact on real-time performance.
(4) The proposed LFD-YOLO network shows improvements in *mAP*_0.5_, F-measure, and *I_r_* by 6.5%, 6.4%, and 8.9%, respectively (92.8% → 96.2%, 90.3% → 96.7%, 87.9% → 98.8%), while the FPS decreases by 3.7 f·s^−1^ (29.1 f·s^−1^ → 25.4 f·s^−1^). This demonstrates that LFD-YOLO significantly enhanced the accuracy of ATB surface defect detection, but moderately impacted real-time performance.

In summary, the substitution of LDconv for CBS, the addition of the DAT deformable attention mechanism, and the optimization of the bounding box loss function with Focaler-IoU in the proposed LFD-YOLO network all effectively improved the accuracy of ATB surface defect detection, with only minor impacts on real-time performance.

### 4.3. Effectiveness in Practical Applications

**Figure 10:**
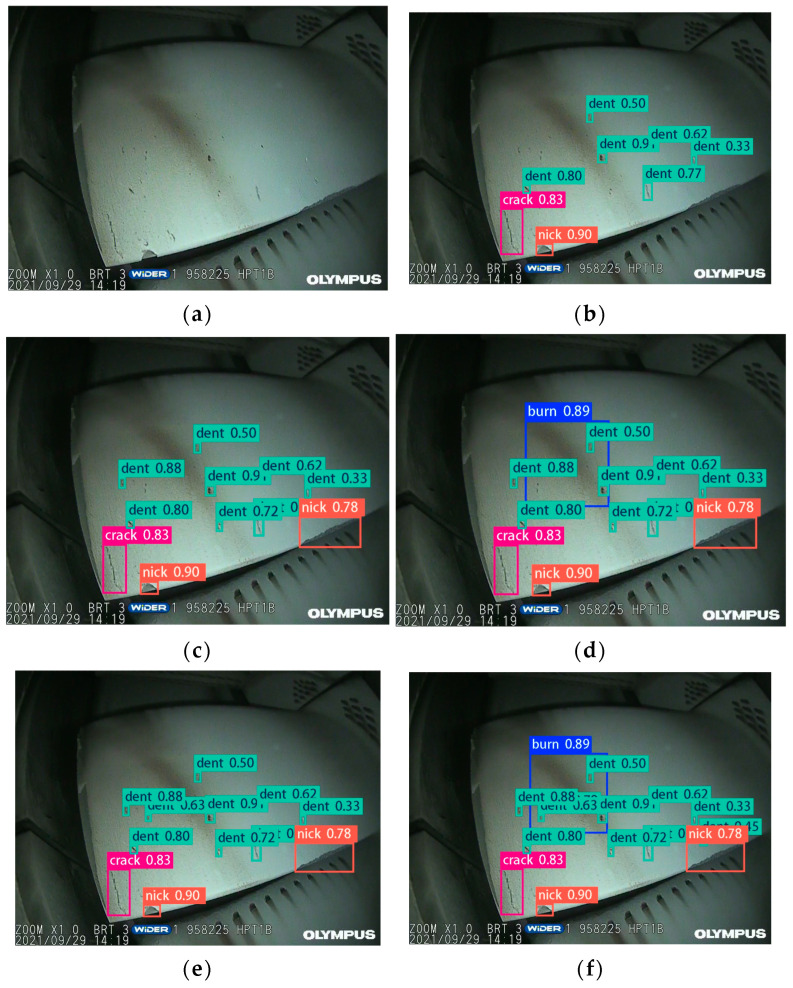
Images of the ATB surface defect detection using ablation experimental networks: (**a**) original image, (**b**) YOLOv8, (**c**) YOLOv8 + LDconv, (**d**) YOLOv8 + DAT, (**e**) YOLOv8 + Focaler-CIoU, and (**f**) LFD-YOLO.

Figure 10 presents the ATB surface defect detection results for each ablation experiment and network. Figure 10a corresponds to the original image, while Figure 10b–f represent the detection results of the YOLOv8, YOLOv8 + LDconv, YOLOv8 + DAT, YOLOv8 + Focaler-CIoU, and LFD-YOLO networks, respectively. Table 5 presents the defect statistics for the number of defects identified by each ablation experiment network. Manual inspection reveals 11 instances of dents, 2 instances of nicks, 1 crack, and 1 burn.

From Figure 10 and Table 5, the following conclusions are made:

(1) The YOLOv8, YOLOv8 + LDconv, and YOLOv8 + Focaler-CIoU networks can only identify three types of defects: dents, nicks, and cracks, detecting 8, 11, and 12 instances, respectively. These networks exhibit missed detections, with burns not being detected. The cumulative accuracy rates for the four types of defects are 53.3%, 73.3%, and 80%, respectively.
(2) The YOLOv8 + DAT network identifies all four types of defects (dents, nicks, cracks, and burns), detecting a total of 13 instances. Although it missed some defects, the cumulative accuracy rate for the four types of defects is 86.7%.
(3) The LFD-YOLO network also identifies all four types of defects (dents, nicks, cracks, and burns), detecting a total of 15 instances with no missed detections. The results align with manual inspections, achieving an accuracy rate of 100%.

In summary, as stepwise improvements to the networks are made, the recognition accuracy for ATB surface defects continues to increase, and the number of detectable ATB surface defects also rises. The proposed LFD-YOLO network was capable of identifying all the defects, demonstrating optimal defect recognition and localization accuracy and satisfactory real-time performance, thereby meeting the on-site detection requirements.

### 4.4. Model Generalization Experiment

**Figure 11:**
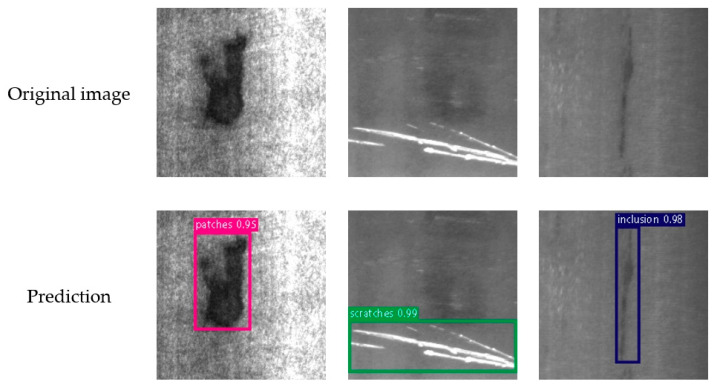
Visualization of the generalization experiment results.

To validate the generalizability of the proposed LFD-YOLO network, supplementary experiments were conducted on the public dataset NEU-DET [50]. The NEU-DET dataset, widely used for surface defect detection, includes six common types of steel surface defects: crazing, inclusion, patches, pitted surface, rolled-in scale, and scratches. The dataset consists of 1800 grayscale images, with 300 samples for each defect type, and each image has a resolution of 200 × 200 pixels. Table 6 presents the comparison of generalization experiment results, and Figure 11 visualizes the generalization experiment results.

It can be observed that the proposed LFD-YOLO achieves *mAP*_0.5_, F-measure, and *I_r_* metrics of 92.2%, 93.7%, and 91.9%, respectively, on the NEU-DET dataset. Although these metrics show a slight decrease compared to those on the ATB dataset, they still reach a high level. The results demonstrate the generalizability and robustness of LFD-YOLO in detecting different types of surface defects.

## 5. Conclusions and Future Work

(1) This paper proposes an ATB surface defect detection method based on LFD-YOLO, which meets the requirements of ATB surface defect detection. The proposed model effectively addresses the challenges of low accuracy and inadequate efficiency in ATB surface defect detection. The method builds upon YOLOv8, which was modified by substituting LDconv for the CBS module, incorporates the DAT deformable attention mechanism, and optimizes the bounding box loss function with Focaler-IoU. In the LFD-YOLO network, the LDconv module replaces the original CBS module in the backbone section, and the DAT deformable attention mechanism is added to improve the detection capability of tiny defects. In the neck section, the PAN–FPN structure is added and combined with the LDconv + DAT deformable attention mechanism to fuse multiscale features and improve the detection of various types of ATB surface defects. The head section employs Focaler-CIoU which optimizes the bounding box loss function, thus also contributing to improved bounding box regression accuracy.
(2) The ablation experimental data confirm that replacing CBS with LDconv improves the network’s *mAP*_0.5_, F-measure, and *I_r_* by 3.1%, 2.9%, and 5.1%, respectively, compared to YOLOv8, while the FPS decreases by 1.9 f·s^−1^. Incorporating the DAT deformable attention mechanism improves the network’s *mAP*_0.5_, F-measure, and *I_r_* by 4.8%, 4.9%, and 8.4%, respectively, compared to YOLOv8, with the FPS decreasing by 1.5 f·s^−1^. Optimizing the bounding box loss function with Focaler-IoU increases the network’s *mAP*_0.5_, F-measure, and *I_r_* by 2.4%, 1.2%, and 3.8%, respectively, compared to YOLOv8, while the FPS decreases by 1.0 f·s^−1^. The proposed LFD-YOLO network improved *mAP*_0.5_, F-measure, and *I_r_* by 6.5%, 6.4%, and 8.9%, respectively, compared to YOLOv8, with the FPS decreasing by 3.7 f·s^−1^. Overall, the results indicate that LFD-YOLO significantly increases the accuracy of ATB surface defect detection, although with a moderate impact on real-time performance.
(3) Comparative experiments and practical application results demonstrate that the proposed LFD-YOLO network outperforms SSD, Faster R-CNN, Mask R-CNN, YOLOv3, YOLOv5, YOLOv7, YOLOv7-tiny, and YOLOv8 in terms of accuracy for ATB surface defect detection. YOLOv8, YOLOv8 + LDconv, and YOLOv8 + Focaler-CIoU can only identify three types of defects (dents, nicks, and cracks), with some missed detections and burns not being detected. YOLOv8 + DAT can identify all four types of defects (dents, nicks, cracks, and burns), but still fails to detect some defects. In contrast, LFD-YOLO can identify all four types of defects—dents, nicks, cracks, and burns—with no missed detections. As network improvements are added incrementally, the recognition accuracy for ATB surface defects continues to increase. The LFD-YOLO network achieves optimal defect recognition accuracy, demonstrates satisfactory real-time performance, and meets the requirements for on-site detection.

Although the proposed method achieves better detection accuracy than the YOLOv8 network, its detection speed decreased. Future work could focus on optimizing the network through lightweight processing. Additionally, experiments on ATB defect detection under complex backgrounds are needed, along with further expansion of the dataset, to facilitate earlier industrialization and the identification of more defect types.

## Figures and Tables

**Table 1:** Quantitative analysis of the different networks.

Model	Backbone	*mAP_0_*_.5_, %	F-Measure, %	FPS, (f·s^−1^)
SSD [43]	VGG-16	61.2	60.9	35.6
Faster R-CNN [28]	ResNet-50	73.7	74.1	16.2
Mask R-CNN [30]	ResNet-50	76.3	78.2	19.3
YOLOv3 [44]	DarkNet-53	77.5	76.7	27.2
YOLOv5 [16]	CSP-Darknet53	86.8	86.9	27.7
YOLOv7 [18]	DarkNet-53 + E-ELAN	89.1	88.7	31.1
YOLOv7-tiny [45]	DarkNet-53 + E-ELAN	77.1	76.8	47.8
YOLOv8 [19]	DarkNet53 + C2f	89.7	90.3	29.1
YOLOv11 [46]	DarkNet53 + C3k2	91.4	91.9	30.7
LFD-YOLO	DarkNet53 + C2f +DAT	96.2	96.7	25.4

**Table 2:** Results of ablation study I.

Model	*mAP*_0.5_, %	F-Measure, %	*I_r_*, %	FPS, (f·s^−1^)
YOLOv8 + LDconv + DAT	93.0	92.4	92.3	27.0
YOLOv8 + LDconv + DAT + EIoU	93.1	91.8	93.2	26.6
YOLOv8 + LDconv + DAT + WIoU	93.5	92.4	92.7	26.3
YOLOv8 + LDconv + DAT + MPDIoU	94.9	94.6	95.1	24.7
LFD-YOLO	96.2	96.7	98.8	25.4

**Table 3:** Results of ablation study II.

Model	*mAP*_0.5_, %	F-Measure, %	*I_r_*, %	FPS, (f·s^−1^)
YOLOv8 + LDconv + Focaler-CIoU	93.2	93.7	94.0	27.1
YOLOv8 + LDconv + Focaler-CIoU+ DAT (backbone)	94.7	94.9	95.2	26.9
YOLOv8 + LDconv + Focaler-CIoU+ DAT (neck)	95.1	94.5	96.0	26.4
LFD-YOLO	96.2	96.7	98.8	25.4

**Table 4:** Results of ablation study III.

Model	*mAP*_0.5_, %	F-Measure, %	*I_r_*, %	FPS, (f·s^−1^)
YOLOv8	89.7	90.3	87.9	29.1
YOLOv8 + LDconv	92.8	93.2	93.0	27.2
YOLOv8 + DAT	94.5	95.2	96.3	27.6
YOLOv8 + Focaler-CIoU	92.1	91.5	91.7	28.1
LFD-YOLO	96.2	96.7	98.8	25.4

**Table 5:** Statistics on the identified surface defects using ablation experimental networks: YOLOv8, YOLOv8 + LDconv, and YOLOv8 + Focaler-CIoU.

Model	Dents	Nicks	Cracks	Burns
YOLOv8	6	1	1	0
YOLOv8 + LDconv	8	2	1	0
YOLOv8 + Focaler-CIoU	9	2	1	0
YOLOv8 + DAT	9	2	1	1
LFD-YOLO	11	2	1	1

**Table 6:** Comparison of the generalization experiment results.

Dataset	*mAP*_0.5_, %	F-Measure, %	*I_r_*, %
NEU-DET [50]	92.2	93.7	91.9
ATB	96.2	96.7	95.2

## Data Availability

The raw data supporting the conclusions of this article will be made available by the authors upon request.
